# Genome-wide analysis of family-1 UDP glycosyltransferases (UGT) and identification of *UGT* genes for FHB resistance in wheat (*Triticum aestivum* L.)

**DOI:** 10.1186/s12870-018-1286-5

**Published:** 2018-04-19

**Authors:** Yi He, Dawood Ahmad, Xu Zhang, Yu Zhang, Lei Wu, Peng Jiang, Hongxiang Ma

**Affiliations:** 1 Institute of Food Crops, Jiangsu Academy of Agricultural Sciences / Jiangsu Collaborative Innovation Center for Modern Crop Production, Nanjing, China; 2 Institute of Biotechnology and Genetic Engineering, The University of Agriculture, Peshawar, Pakistan

**Keywords:** UDP-glycosyltransferase, *Fusarium* head blight, Wheat, Expression pattern, Phylogeny, Deoxynivalenol

## Abstract

**Background:**

*Fusarium* head blight (FHB), a devastating disease in wheat worldwide, results in yield loses and mycotoxin, such as deoxynivalenol (DON), accumulation in infected grains. DON also facilitates the pathogen colonization and spread of FHB symptoms during disease development. UDP-glycosyltransferase enzymes (UGTs) are known to contribute to detoxification and enhance FHB resistance by glycosylating DON into DON-3-glucoside (D3G) in wheat. However, a comprehensive investigation of wheat (*Triticum aestivum*) *UGT* genes is still lacking.

**Results:**

In this study, we carried out a genome-wide analysis of family-1 UDP glycosyltransferases in wheat based on the PSPG conserved box that resulted in the identification of 179 putative *UGT* genes. The identified genes were clustered into 16 major phylogenetic groups with a lack of phylogenetic group K. The *UGT* genes were invariably distributed among all the chromosomes of the 3 genomes. At least 10 intron insertion events were found in the *UGT* sequences, where intron 4 was observed as the most conserved intron. The expression analysis of the wheat *UGT* genes using both online microarray data and quantitative real-time PCR verification suggested the distinct role of *UGT* genes in different tissues and developmental stages. The expression of many *UGT* genes was up-regulated after *Fusarium graminearum* inoculation, and six of the genes were further verified by RT-qPCR.

**Conclusion:**

We identified 179 *UGT* genes from wheat using the available sequenced wheat genome. This study provides useful insight into the phylogenetic structure, distribution, and expression patterns of family-1 UDP glycosyltransferases in wheat. The results also offer a foundation for future work aimed at elucidating the molecular mechanisms underlying the resistance to FHB and DON accumulation.

**Electronic supplementary material:**

The online version of this article (10.1186/s12870-018-1286-5) contains supplementary material, which is available to authorized users.

## Background

*Fusarium* head blight (FHB) or scab, primarily caused by *Fusarium graminearum*, is one of the most devastating diseases in wheat and barley worldwide [1]. FHB infection not only results in heavy yield loss but also deteriorates grain quality due to the production of trichothecene mycotoxins such as deoxynivalenol (DON), nivalenol (NIV) and their acetylated forms 3 (or 15)-acetyl-4-deoxynivalenol, 4-acetylnivalenol or fusarenone X (FX), of which DON is one of the most important members [2]. The accumulation of DON and other toxins in the infected grains is making wheat unfit for human and livestock consumption posing a risk to world food security [3]. DON along with other trichothecenes also act as fungal virulence factors, facilitating the colonization and spread of scab symptoms within wheat spikes [2]. When the *TRI5* gene was disrupted, the DON-nonproducing mutants of *F. graminearum* lacked the ability to spread in wheat spikes [4]. The resistance to DON accumulation is different in FHB infected wheat varieties, highlighting the involvement of a host resistance system during the course of DON production [1]. DON-3-glucoside (D3G), a non-toxic masked form of DON, has been observed in wheat grains in addition to DON and is stored in plant cell vacuoles [5]. Using their enzymatic defense system, plants convert DON into D3G as previously described for the first time in Arabidopsis [6]. The resistance to FHB infection and D3G accumulation in wheat grains is correlated with the ability of a higher conversion of DON into D3G [7]. DON conversion into D3G, a natural detoxification process in plants called glycosylation, along with other mechanisms of detoxification such as acetylation and de-epoxidation, reduce mycotoxin accumulation and are potential resistance factors against FHB [2, 5, 8].

Glycosylation is a widespread cellular modification reaction in all living organisms, attaching a carbohydrate to the hydroxyl or other functional group of a molecule in a biosynthetic pathway [9]. Glycosylation is a form of co-translational and post-translational modification altering the chemical property, sub-cellular location and activity of a variety of bio-molecules [10]. Glycosylation modifications are catalyzed by glycosyltransferase enzymes (GTs), which are highly divergent, polyphyletic and belong to a multigene family [11]. Glycosylation, in addition to other reactions, paves the way to the production of a huge variety of secondary metabolites in plants. GTs from different species can be classified into 94 families based on their amino acid sequence similarities, catalytic mechanisms as well as the presence of conserved sequence motifs according to the most recent update of CAZy (http://www.cazy.org/GlycosylTransferases.html). Among them, family-1 GTs are the most common glycosyltransferases in the plant kingdom generally catalyzing the process of glycosylation by transferring sugar moieties from activated donor molecules to acceptor molecules [5, 12, 13]. Family-1 GTs use uridine 5′-diphosphate sugars as the donor molecule; hence, they are also named uridine-diphosphate glycosyltransferases (UGTs) [14]. These enzymes act upon a huge variety of highly diverse and complex substrates in the plant kingdom such as flavonoids, terpenes, auxin, cytokinin, salicylic acid and many others to regulate plant growth, development, disease resistance and interaction with the environment [15]. The sequences at N-terminal region of these enzymes are highly diverse and are considered to be responsible for recognition of a variety of substrates. The C-terminal region on the other hand contains a conserved motif called Plant Secondary Product Glycosyltransferase (PSPG). The PSPG box is a unique, well conserved region of 44 amino acids found in all UGTs across all studied plant taxa [14, 16].

UDP-glycosyltransferases have been identified in various plant species, including *Linum usitatissimum*, *Arabidopsis thaliana*, *Malus domestica*, *Vitis vinifera*, *Populus trichocarpa*, *Glycine max*, *Mimulus guttatus*, *Oryza sativa*, *Sorghum bicolor*, *Brachypodium distachyon*, *Zea mays*, *Gossypium raimondii*, *Gossypium arboreum, Gossypium hirsutum* and most recently in *Prunus persica*, *Brassica rapa*, and *Brassica oleracea* with approximately more than 100 UGTs in all the higher plants investigated [15, 17–22]. UGTs have been shown to display broad as well as selective substrate specificity in plants recognizing a wide range of acceptor molecules [23, 24]. The UGTs not only glycosylate acceptor molecules, such as anthocyanidins, flavonols, flavonoids, saponins, sterols terpenoids, phenylpropanoids and plant hormones, but also detoxify and deactivate xenobiotics and play a pivotal role in plant-pathogen interactions [9, 21].

Functioning of UGTs has been described in many plants such as Arabidopsis, kiwifruit and strawberry [25–28]. Besides their various other attributes, *UGT* genes have also been demonstrated to contribute to FHB resistance in crops possibly through DON glycosylation [5]. Four highly DON inducible candidate GTs were identified in barley and one of them *HvUGT13248* gene, the first monocot DON-glucosyltransferase, extended DON resistance in yeast and has since been expressed in Arabidopsis and wheat providing resistance against DON and other mycotoxins [29–33]. Similarly, two DON detoxification genes conjugating DON to D3G have been identified in *Brachypodium distachyon* [19]. Recently the *HvUGT-10 W1* gene isolated from an FHB resistant barley variety (10 W1) also conferred FHB tolerance [34]. In wheat only a few candidate *UGT* genes such as *TaUGTB2*, *TaUGT1*, *TaUGT2*, *TaUGT3*, *TaUGT4*, and *TaUGT12887* have been identified and the later 4 genes have been shown to be closely related to FHB resistance; however, a large scale systematic investigation of UGTs in the wheat genome is still lacking [35–39].

In this study we identified 179 *UGT* genes from wheat using the available sequenced wheat genome. The genetic relationships of these UGTs were determined using sequence alignment and phylogenetic tree analysis. The differential expression of genes in various wheat tissues as well as wheat spikes treated with *F. graminearum* vs control expressions patterns were analyzed using NCBI and universal microarray data and further confirmed through RT-qPCR analysis of the selected genes. This study will hopefully provide a baseline to conduct future functional characterization analysis of wheat *UGT* genes in order to understand the molecular mechanisms behind substrate specificity in general and especially the resistance to FHB and DON accumulation in crops.

## Methods

### Identification of *UGT* genes in wheat

The 44-amino acid conserved sequence of the PSPG motif was used as a query to search against the wheat genome database (TGACv1) at the Ensemble Plants (http://plants.ensembl.org/Triticum_aestivum/) by HMMER profile to identify members of the UGT protein family in wheat [9, 40]. The identified protein sequences of each UGT were further verified through the PFAM (http://pfam.xfam.org/) and the SMART (http://smart.embl-heidelberg.de/) databases to confirm the presence of the UDP-glycosyltransferase domain. The information (including amino acid length, transcript count and location) was also obtained from Ensemble Plants. The molecular weight (MW) and isoelectric point (PI) of each UGT protein were calculated using the online ExPASy program (http://web.expasy.org/compute_pi/) [41]. The subcellular localization of each UGT protein was predicted using the online CELLO v2.5 system (http://cello.life.nctu.edu.tw/cello.html) [42].

### Sequence alignment and phylogenetic analysis

Multiple sequence alignments of the wheat UGT protein sequences were performed by MUSCLE (http://www.ebi.ac.uk/Tools/msa/muscle/). The phylogenetic tree was constructed using MEGA 7.0 software (http://www.megasoftware.net/) based on the full-length UGT protein sequences through a neighbor-joining algorithm using a 1000 bootstrap value [43].

### Chromosomal locations

The genetic location of each UGT on the chromosomes was retrieved from the final TGACv1 map [40]. The genetic map of each UGT on the chromosome was modified from the primary map drawn by the MapInspect tool (http://mapinspect.software.informer.com/).

### Intron mapping

The wheat *UGT* intron map was constructed by determining the intron positions, splice sites and phases. The intron phases were determined as described previously: the introns positioned between two triplet codons were defined as phase 0, introns positioned after the first base in the codon were defined as phase 1, and the introns positioned after the second base in the codon were defined as phase 2 [20].

### Expression profile analysis

A genome-wide expression analysis of the wheat *UGT* genes in various organs and developmental stages was performed using high-throughput RNA sequence data from 5 organs (root, stem, leaf, spike and grain), each with 3 developmental stages (https://urgi.versailles.inra.fr/files/RNASeqWheat/) [44]. To analyze the expression profiles of the wheat *UGT* genes after *F. graminearum* inoculation, the Affymetrix wheat array data with wheat spikelets from the susceptible spring wheat cultivar Roblin inoculated with either water or *F. graminearum* strains that do or do not produce DON were downloaded (https://www.ncbi.nlm.nih.gov/geo/) for further analysis (accession number GSE54554). The expression profiles of these *UGT* genes were generated by using MeV 4.7 software (https://sourceforge.net/projects/mev-tm4/files/mev-tm4/).

### Plant materials and treatment

The wheat cultivar used in this study was Annong 8455, which is highly susceptible to FHB in China. The wheat plants were grown in a green house at 22 °C for 14-h light and 12 °C for 10-h dark at the Jiangsu Academy of Agricultural Sciences, China.

The early anthesis stage spikelets were chosen for further *F. graminearum* or water (CK) inoculation as described previously [45]. Approximately 10 μl of fungal suspension (1 × 10^6^ conidia per mL) of *F. graminearum* or water was injected into the central spikelet of a spike during early anthesis. The inoculated spikes were covered with a plastic bag for 3 days.

### RNA isolation and RT-qPCR analysis

To validate the expression pattern of the six selected genes, the total RNA was isolated from the root, stem, leaf, pre-emergence spikelet, pre-anthesis spikelet, and grains of 7, 14 and 21 days after flowering (DAF) using the Promega SV total RNA isolation system (Promega, United States), according to the manufacturer’s instructions. The RNA of the spikelets treated with water or *F. graminearum* after 2 and 4 days was also extracted in the same way. The first-strand cDNAs were synthesized from the total RNA by using the PrimeScript 1st strand cDNA Synthesis Kit (Takara Bio, Dalian, China), according to the manufacturer’s manual. Real-time PCR was performed with a Roche thermal cycler 96 using SYBR Green to detect gene expression. The wheat *tubulin* gene was used as an endogenous control. The gene specific primers used for RT-qPCR are listed in the Additional file 1: Table S1. The PCR conditions were as follows: 95 °C for 30 s, 95 °C for 5 s, 60 °C for 20 s and 72 °C for 10 s for 45 cycles. Data from the individual runs were collated using the 2^−ΔΔCT^ method [46]. All the reactions were performed using at least three replicates.

## Results

### Identification of *UGT* genes in wheat

A nearly complete and accurate sequence assembly of the allohexaploid wheat reference accession, Chinese Spring (CS42), was released recently, enabling a more in-depth analysis of *UGT* genes in this key global crop [40]. The conserved UGT domain of 44 amino acids called the PSPG box was used in this study to identify the presence of *UGT* genes in the wheat genome [9]. A total of 179 putative *UGT* genes having an average length of 471 amino acids were identified and used for further analysis (Table 1). Most of the genes were in the range of 400 to 500 amino acids, while only a few genes were above 500 and below 300 amino acids in size and 1 to 3 transcripts were counted for these genes (Additional file 2: Figure S1). The theoretical isoelectric point (pI) and molecular weight (Mw) ranged from 4.77 to 10.32 (average pI = 5.79) and from 26.9 kDa to 76.5 kDa (average Mw = 50.9 kDa), respectively (Table 1). The theoretical cellular localization showed 22, 19, 5, 2 and 0.6% of the genes were localized only into the chloroplast, cytoplasm, plasma membrane, mitochondria and nucleus, respectively, while the rest of the genes were localized into any of these compartments (Table 1).

**Table 1:** The list of the putative wheat *UGT* genes identified in this study

No.	Gene stable ID	Amino acid length	Transcript count	PI	MW (kDa)	Subcellular location	Location
1	TRIAE_CS42_1AL_TGACv1_000152_AA0004850	569	2	5.6	62.3	Chloroplast Cytoplasmic	TGACv1_000152_1AL: 34,636–36,800
2	TRIAE_CS42_1AL_TGACv1_000696_AA0017290	479	1	5.7	52.2	Cytoplasmic	TGACv1_000696_1AL: 81,630–83,635
3	TRIAE_CS42_1AL_TGACv1_001147_AA0025960	456	1	5.3	49.3	Chloroplast Cytoplasmic PlasmaMembrane	TGACv1_001147_1AL: 8789–10,755
4	TRIAE_CS42_1AL_TGACv1_001208_AA0027000	474	1	6	50.6	Mitochondrial PlasmaMembrane	TGACv1_001208_1AL: 53,142–54,992
5	TRIAE_CS42_1AL_TGACv1_001652_AA0033630	324	1	6.2	35	PlasmaMembrane	TGACv1_001652_1AL: 54,716–56,210
6	TRIAE_CS42_1AS_TGACv1_020318_AA0076580	505	3	5.3	55.2	Cytoplasmic Mitochondrial	TGACv1_020318_1AS: 8106–11,995
7	TRIAE_CS42_1BL_TGACv1_031128_AA0108300	464	1	5.4	49.6	PlasmaMembrane	TGACv1_031128_1BL: 73,386–75,090
8	TRIAE_CS42_1BL_TGACv1_032253_AA0127550	504	1	6	54.1	Chloroplast Cytoplasmic	TGACv1_032253_1BL: 30,766–32,648
9	TRIAE_CS42_1BL_TGACv1_032609_AA0132000	451	1	6	48.4	PlasmaMembrane	TGACv1_032609_1BL: 18,947–20,825
10	TRIAE_CS42_1BL_TGACv1_034076_AA0143330	423	1	5.9	45.3	Cytoplasmic Mitochondrial	TGACv1_034076_1BL: 13,111–14,941
11	TRIAE_CS42_1BS_TGACv1_049891_AA0163670	536	1	5.5	58	Cytoplasmic Chloroplast	TGACv1_049891_1BS: 3226–6480
12	TRIAE_CS42_1BS_TGACv1_050208_AA0169040	542	1	5.6	56.7	Chloroplast PlasmaMembrane	TGACv1_050208_1BS: 22,661–24,740
13	TRIAE_CS42_1DL_TGACv1_061249_AA0190020	486	1	6	52.4	Cytoplasmic PlasmaMembrane	TGACv1_061249_1DL: 118,022–119,855
14	TRIAE_CS42_1DL_TGACv1_061472_AA0196220	473	1	5.6	50.6	Cytoplasmic	TGACv1_061472_1DL: 36,915–38,810
15	TRIAE_CS42_1DL_TGACv1_061688_AA0201770	386	1	6	42.6	Cytoplasmic	TGACv1_061688_1DL: 38,929–45,276
16	TRIAE_CS42_1DL_TGACv1_062127_AA0209080	497	1	5.2	53	Chloroplast Cytoplasmic	TGACv1_062127_1DL: 35,096–37,034
17	TRIAE_CS42_2AL_TGACv1_092977_AA0268460	496	2	5.9	53.3	Chloroplast PlasmaMembrane	TGACv1_092977_2AL: 132,556–134,743
18	TRIAE_CS42_2AL_TGACv1_094039_AA0291500	363	1	6.7	37.6	Chloroplast PlasmaMembrane	TGACv1_094039_2AL: 11,847–13,415
19	TRIAE_CS42_2AL_TGACv1_094526_AA0299210	444	1	5.5	47.4	PlasmaMembrane Chloroplast Cytoplasmic	TGACv1_094526_2AL: 10,962–12,595
20	TRIAE_CS42_2AL_TGACv1_094669_AA0301250	493	1	5.7	53.5	Chloroplast PlasmaMembrane Cytoplasmic	TGACv1_094669_2AL: 43,325–45,075
21	TRIAE_CS42_2AL_TGACv1_095609_AA0312870	479	1	5.4	51.7	Cytoplasmic	TGACv1_095609_2AL: 23,136–25,080
22	TRIAE_CS42_2AS_TGACv1_112708_AA0343800	465	1	9	51	Mitochondrial Chloroplast	TGACv1_112708_2AS: 5716–7407
23	TRIAE_CS42_2AS_TGACv1_113114_AA0351430	314	1	5	35.1	Cytoplasmic	TGACv1_113114_2AS: 82,126–83,619
24	TRIAE_CS42_2AS_TGACv1_113164_AA0352370	474	1	5.5	51.6	Chloroplast	TGACv1_113164_2AS: 56,381–58,985
25	TRIAE_CS42_2AS_TGACv1_113792_AA0360520	471	1	5.8	51.1	Chloroplast	TGACv1_113792_2AS: 1565–3515
26	TRIAE_CS42_2AS_TGACv1_113792_AA0360550	470	1	5.4	50.6	Cytoplasmic Chloroplast	TGACv1_113792_2AS: 12,613–15,275
27	TRIAE_CS42_2BL_TGACv1_132343_AA0436900	489	1	5.7	53.4	Chloroplast	TGACv1_132343_2BL: 30,155–32,015
28	TRIAE_CS42_2BL_TGACv1_133391_AA0442380	485	1	5.4	51.3	Chloroplast PlasmaMembrane Cytoplasmic	TGACv1_133391_2BL: 9937–11,795
29	TRIAE_CS42_2BS_TGACv1_146052_AA0454210	480	2	5.4	54.9	Cytoplasmic Chloroplast	TGACv1_146052_2BS: 172,038–174,105
30	TRIAE_CS42_2BS_TGACv1_146052_AA0454220	234	1	5.2	26	Chloroplast Cytoplasmic	TGACv1_146052_2BS: 175,207–176,915
31	TRIAE_CS42_2BS_TGACv1_146119_AA0455760	477	1	5.9	51.6	Chloroplast PlasmaMembrane	TGACv1_146119_2BS: 73,276–75,131
32	TRIAE_CS42_2BS_TGACv1_146212_AA0458920	477	1	5.9	51.8	Chloroplast	TGACv1_146212_2BS: 40,106–41,900
33	TRIAE_CS42_2BS_TGACv1_146276_AA0461240	458	1	6.2	49.8	Chloroplast	TGACv1_146276_2BS: 43,496–45,300
34	TRIAE_CS42_2BS_TGACv1_146286_AA0461540	466	1	8.4	51.1	Chloroplast Mitochondrial	TGACv1_146286_2BS: 86,006–87,750
35	TRIAE_CS42_2BS_TGACv1_146500_AA0466680	498	1	5.2	52.4	Chloroplast	TGACv1_146500_2BS: 99,495–101,475
36	TRIAE_CS42_2BS_TGACv1_146843_AA0473870	505	1	6.4	53.9	Chloroplast PlasmaMembrane	TGACv1_146843_2BS: 70,557–72,425
37	TRIAE_CS42_2BS_TGACv1_147441_AA0483230	477	1	6.1	50.8	Cytoplasmic	TGACv1_147441_2BS: 41,416–43,450
38	TRIAE_CS42_2BS_TGACv1_147641_AA0485890	519	1	5.6	56.9	Chloroplast	TGACv1_147641_2BS: 19,336–29,770
39	TRIAE_CS42_2DL_TGACv1_158399_AA0517610	492	1	5.4	54	Cytoplasmic	TGACv1_158399_2DL: 87,645–89,545
40	TRIAE_CS42_2DL_TGACv1_159414_AA0537930	482	1	5.4	51.9	PlasmaMembrane Cytoplasmic	TGACv1_159414_2DL: 9459–11,225
41	TRIAE_CS42_2DL_TGACv1_159743_AA0542200	469	1	5.5	51.3	Cytoplasmic	TGACv1_159743_2DL: 29,369–31,464
42	TRIAE_CS42_2DL_TGACv1_160147_AA0547510	499	1	5.9	53.3	PlasmaMembrane Chloroplast	TGACv1_160147_2DL: 11,782–13,675
43	TRIAE_CS42_2DL_TGACv1_160383_AA0549920	485	2	5.5	53.7	Cytoplasmic Mitochondrial Chloroplast	TGACv1_160383_2DL: 17,866–19,990
44	TRIAE_CS42_2DL_TGACv1_160484_AA0550940	476	1	6.4	51.5	PlasmaMembrane	TGACv1_160484_2DL: 22,697–24,835
45	TRIAE_CS42_2DS_TGACv1_177189_AA0568300	505	1	5	53.5	Chloroplast	TGACv1_177189_2DS: 168,676–170,825
46	TRIAE_CS42_2DS_TGACv1_177304_AA0572860	462	1	5.2	49.6	PlasmaMembrane Cytoplasmic	TGACv1_177304_2DS: 18,887–22,885
47	TRIAE_CS42_2DS_TGACv1_177710_AA0582890	508	2	5.4	55.8	Chloroplast	TGACv1_177710_2DS: 37,388–39,565
48	TRIAE_CS42_2DS_TGACv1_177916_AA0587150	493	1	5.7	53.3	Chloroplast	TGACv1_177916_2DS: 19,783–21,976
49	TRIAE_CS42_2DS_TGACv1_178033_AA0589680	467	1	6.4	50.9	Chloroplast	TGACv1_178033_2DS: 37,145–39,412
50	TRIAE_CS42_2DS_TGACv1_178118_AA0591100	372	1	8.5	40	PlasmaMembrane	TGACv1_178118_2DS: 56,233–58,495
51	TRIAE_CS42_2DS_TGACv1_178131_AA0591440	497	1	5.7	53	Chloroplast PlasmaMembrane Cytoplasmic	TGACv1_178131_2DS: 31,516–33,382
52	TRIAE_CS42_2DS_TGACv1_178315_AA0594020	476	1	6	51.3	Chloroplast	TGACv1_178315_2DS: 49,188–50,874
53	TRIAE_CS42_2DS_TGACv1_178795_AA0601130	469	1	6.3	50	Cytoplasmic	TGACv1_178795_2DS: 43,800–45,517
54	TRIAE_CS42_3AL_TGACv1_194443_AA0633160	500	2	5.6	54.5	Cytoplasmic	TGACv1_194443_3AL: 22,846–28,150
55	TRIAE_CS42_3AL_TGACv1_194677_AA0637610	475	1	5.5	51.5	Cytoplasmic Chloroplast	TGACv1_194677_3AL: 53,009–55,235
56	TRIAE_CS42_3AL_TGACv1_194875_AA0641170	466	1	5.7	50.6	Cytoplasmic Chloroplast	TGACv1_194875_3AL: 63,326–65,525
57	TRIAE_CS42_3AS_TGACv1_210937_AA0681620	414	1	5.3	44.4	Chloroplast Cytoplasmic	TGACv1_210937_3AS: 119,816–121,716
58	TRIAE_CS42_3AS_TGACv1_211248_AA0687180	551	1	5	59.3	Cytoplasmic Chloroplast	TGACv1_211248_3AS: 47,048–49,241
59	TRIAE_CS42_3AS_TGACv1_211655_AA0692640	472	1	5.3	50.6	Cytoplasmic	TGACv1_211655_3AS: 41,556–44,305
60	TRIAE_CS42_3AS_TGACv1_211823_AA0694680	511	1	5.5	56.9	Cytoplasmic	TGACv1_211823_3AS: 30,616–32,748
61	TRIAE_CS42_3AS_TGACv1_211823_AA0694700	492	1	5.4	54.7	Cytoplasmic	TGACv1_211823_3AS: 44,976–46,896
62	TRIAE_CS42_3B_TGACv1_220919_AA0723700	472	1	6.1	51.3	Chloroplast	TGACv1_220919_3B: 29,856–31,633
63	TRIAE_CS42_3B_TGACv1_220919_AA0723750	469	1	5.5	50.8	Chloroplast PlasmaMembrane	TGACv1_220919_3B: 149,148–150,855
64	TRIAE_CS42_3B_TGACv1_221277_AA0735990	473	1	5.1	51	Cytoplasmic Chloroplast	TGACv1_221277_3B: 36,097–37,935
65	TRIAE_CS42_3B_TGACv1_221877_AA0752320	496	1	5.4	54.8	Cytoplasmic	TGACv1_221877_3B: 70,264–73,165
66	TRIAE_CS42_3B_TGACv1_221924_AA0753300	468	1	5.4	50.8	Cytoplasmic Chloroplast	TGACv1_221924_3B: 38,292–39,921
67	TRIAE_CS42_3B_TGACv1_222356_AA0762980	464	1	5.3	50.9	PlasmaMembrane Chloroplast Cytoplasmic	TGACv1_222356_3B: 55,147–57,245
68	TRIAE_CS42_3B_TGACv1_223815_AA0787850	461	1	5.2	50	Chloroplast Cytoplasmic	TGACv1_223815_3B: 24,267–25,985
69	TRIAE_CS42_3B_TGACv1_224677_AA0799850	457	2	5.4	49.9	Chloroplast Cytoplasmic Mitochondrial	TGACv1_224677_3B: 12,637–14,485
70	TRIAE_CS42_3B_TGACv1_228792_AA0827590	403	1	5.2	44.8	Cytoplasmic	TGACv1_228792_3B: 13,838–16,085
71	TRIAE_CS42_3DL_TGACv1_249782_AA0856200	465	1	6	50.8	Cytoplasmic	TGACv1_249782_3DL: 45,815–54,365
72	TRIAE_CS42_3DL_TGACv1_249823_AA0856930	481	1	5.4	52.8	Cytoplasmic	TGACv1_249823_3DL: 53,186–55,160
73	TRIAE_CS42_3DL_TGACv1_251186_AA0878520	489	1	6.1	53.1	Chloroplast Mitochondrial	TGACv1_251186_3DL: 6166–8082
74	TRIAE_CS42_3DL_TGACv1_251733_AA0884380	472	1	5.8	51.1	Chloroplast	TGACv1_251733_3DL: 18,147–19,817
75	TRIAE_CS42_3DS_TGACv1_271859_AA0909590	560	1	5.4	51.4	Chloroplast Cytoplasmic Mitochondrial	TGACv1_271859_3DS: 46,873–53,915
76	TRIAE_CS42_3DS_TGACv1_272095_AA0914550	443	1	5.5	48.2	Chloroplast Cytoplasmic	TGACv1_272095_3DS: 18,456–20,253
77	TRIAE_CS42_3DS_TGACv1_272144_AA0915540	380	2	5.8	42.2	Cytoplasmic	TGACv1_272144_3DS: 15,466–17,879
78	TRIAE_CS42_3DS_TGACv1_272561_AA0922330	498	1	5.7	53.7	Cytoplasmic Mitochondrial Chloroplast	TGACv1_272561_3DS: 42,311–44,165
79	TRIAE_CS42_3DS_TGACv1_274000_AA0934260	484	1	5.3	51.8	Cytoplasmic	TGACv1_274000_3DS: 16,535–18,425
80	TRIAE_CS42_4AL_TGACv1_288576_AA0952450	503	2	5.4	56.2	Chloroplast Mitochondrial Cytoplasmic	TGACv1_288576_4AL: 121,655–123,785
81	TRIAE_CS42_4AL_TGACv1_291270_AA0993350	455	1	5.8	47	Chloroplast	TGACv1_291270_4AL: 21,446–23,100
82	TRIAE_CS42_4AL_TGACv1_291728_AA0996300	507	1	4.9	54.4	Chloroplast Cytoplasmic	TGACv1_291728_4AL: 4996–7190
83	TRIAE_CS42_4AL_TGACv1_292113_AA0997730	498	1	8.7	54.5	Mitochondrial	TGACv1_292113_4AL: 17,011–18,815
84	TRIAE_CS42_4AL_TGACv1_292676_AA0999440	506	1	5	54.5	Chloroplast Cytoplasmic	TGACv1_292676_4AL: 10,676–12,740
85	TRIAE_CS42_4AL_TGACv1_293019_AA1000030	452	1	6.6	49.7	Mitochondrial Cytoplasmic	TGACv1_293019_4AL: 8872–10,875
86	TRIAE_CS42_4BL_TGACv1_320707_AA1046800	359	1	5.5	39.7	PlasmaMembrane Cytoplasmic	TGACv1_320707_4BL: 6020–7485
87	TRIAE_CS42_4BS_TGACv1_327950_AA1079620	576	1	8.4	62.3	PlasmaMembrane Mitochondrial	TGACv1_327950_4BS: 204,456–207,103
88	TRIAE_CS42_4BS_TGACv1_329322_AA1100160	455	1	6.1	47.2	Chloroplast	TGACv1_329322_4BS: 51,826–53,691
89	TRIAE_CS42_4BS_TGACv1_329455_AA1101520	461	1	6.5	49.8	Chloroplast Cytoplasmic	TGACv1_329455_4BS: 26,226–28,193
90	TRIAE_CS42_4BS_TGACv1_329471_AA1101760	567	1	7.2	61.6	PlasmaMembrane	TGACv1_329471_4BS: 21,537–26,265
91	TRIAE_CS42_4BS_TGACv1_332581_AA1110480	461	1	5.8	49.5	Chloroplast Cytoplasmic PlasmaMembrane	TGACv1_332581_4BS: 1227–3355
92	TRIAE_CS42_4DL_TGACv1_343563_AA1136610	474	1	5.4	51.3	Chloroplast	TGACv1_343563_4DL: 21,876–23,872
93	TRIAE_CS42_4DL_TGACv1_344211_AA1144960	482	1	5.6	52.4	Chloroplast	TGACv1_344211_4DL: 9685–11,555
94	TRIAE_CS42_5AL_TGACv1_374728_AA1207660	491	1	6	53.4	Cytoplasmic	TGACv1_374728_5AL: 108,276–110,700
95	TRIAE_CS42_5AL_TGACv1_375188_AA1217460	429	1	5.9	46.3	Cytoplasmic Chloroplast PlasmaMembrane	TGACv1_375188_5AL: 37,326–38,987
96	TRIAE_CS42_5AL_TGACv1_375684_AA1225590	475	2	5.8	51.2	Cytoplasmic Chloroplast	TGACv1_375684_5AL: 54,671–56,869
97	TRIAE_CS42_5AL_TGACv1_375893_AA1228550	461	1	6.1	49.4	Mitochondrial	TGACv1_375893_5AL: 47,896–49,580
98	TRIAE_CS42_5AL_TGACv1_375929_AA1229020	476	1	5.6	51.7	PlasmaMembrane	TGACv1_375929_5AL: 57,318–59,915
99	TRIAE_CS42_5AL_TGACv1_376019_AA1230850	491	1	5.8	52.5	Cytoplasmic Mitochondrial	TGACv1_376019_5AL: 40,796–42,980
100	TRIAE_CS42_5AL_TGACv1_377811_AA1249610	472	1	5.8	51.9	Chloroplast	TGACv1_377811_5AL: 10,736–13,180
101	TRIAE_CS42_5BL_TGACv1_404184_AA1288910	490	1	5	53.3	Chloroplast	TGACv1_404184_5BL: 196,689–198,409
102	TRIAE_CS42_5BL_TGACv1_404184_AA1288920	490	1	5.5	53.3	Chloroplast	TGACv1_404184_5BL: 293,989–296,149
103	TRIAE_CS42_5BL_TGACv1_404233_AA1291500	506	1	5.3	54.9	Cytoplasmic	TGACv1_404233_5BL: 154,317–156,325
104	TRIAE_CS42_5BL_TGACv1_404244_AA1291960	470	1	5.6	50.6	Cytoplasmic	TGACv1_404244_5BL: 82,335–84,305
105	TRIAE_CS42_5BL_TGACv1_404293_AA1294180	497	1	5.7	53.7	Cytoplasmic Chloroplast	TGACv1_404293_5BL: 93,246–95,160
106	TRIAE_CS42_5BL_TGACv1_404294_AA1294310	477	1	5.9	50.3	Chloroplast	TGACv1_404294_5BL: 185,737–187,835
107	TRIAE_CS42_5BL_TGACv1_404418_AA1299240	471	1	5.6	50.8	Chloroplast Cytoplasmic	TGACv1_404418_5BL: 207,022–208,895
108	TRIAE_CS42_5BL_TGACv1_405759_AA1334850	464	1	5.8	50.4	Mitochondrial PlasmaMembrane	TGACv1_405759_5BL: 73,581–75,127
109	TRIAE_CS42_5BL_TGACv1_406257_AA1343160	473	1	6.1	51.2	Mitochondrial	TGACv1_406257_5BL: 33,956–36,228
110	TRIAE_CS42_5BL_TGACv1_406579_AA1347330	491	3	5.4	53.3	Chloroplast Cytoplasmic	TGACv1_406579_5BL: 19,551–21,635
111	TRIAE_CS42_5BL_TGACv1_406904_AA1351330	457	1	6.1	49.4	Chloroplast	TGACv1_406904_5BL: 33,027–34,745
112	TRIAE_CS42_5BL_TGACv1_408090_AA1361610	374	1	5.3	40.7	Cytoplasmic	TGACv1_408090_5BL: 6001–9585
113	TRIAE_CS42_5BS_TGACv1_424806_AA1391870	444	1	6.2	48.3	PlasmaMembrane Chloroplast	TGACv1_424806_5BS: 5556–7050
114	TRIAE_CS42_5DL_TGACv1_433291_AA1408500	490	1	5.1	52.4	PlasmaMembrane Chloroplast	TGACv1_433291_5DL: 25,092–26,905
115	TRIAE_CS42_5DL_TGACv1_434244_AA1432540	456	1	4.8	50.3	Chloroplast Cytoplasmic	TGACv1_434244_5DL: 7506–11,405
116	TRIAE_CS42_5DL_TGACv1_435855_AA1455600	491	3	5.4	53.5	Chloroplast Cytoplasmic	TGACv1_435855_5DL: 4016–6117
117	TRIAE_CS42_5DL_TGACv1_436083_AA1457870	455	2	5.6	50	Cytoplasmic	TGACv1_436083_5DL:20522–22,605
118	TRIAE_CS42_5DS_TGACv1_456986_AA1480690	506	3	5.1	54.6	Chloroplast	TGACv1_456986_5DS: 56,363–58,465
119	TRIAE_CS42_5DS_TGACv1_457896_AA1490570	454	1	6.5	49.3	PlasmaMembrane Chloroplast	TGACv1_457896_5DS: 16,778–18,475
120	TRIAE_CS42_6AL_TGACv1_471580_AA1511220	492	1	5.8	53.3	Chloroplast Cytoplasmic	TGACv1_471580_6AL: 48,286–50,301
121	TRIAE_CS42_6AL_TGACv1_472815_AA1526300	492	1	5.2	53.7	Cytoplasmic	TGACv1_472815_6AL: 30,598–36,845
122	TRIAE_CS42_6AL_TGACv1_473165_AA1529140	486	2	5.6	53.1	Chloroplast	TGACv1_473165_6AL: 22,571–24,615
123	TRIAE_CS42_6AS_TGACv1_486256_AA1558890	515	1	5.9	56.4	Chloroplast	TGACv1_486256_6AS: 20,827–22,845
124	TRIAE_CS42_6AS_TGACv1_486559_AA1562640	480	1	5.4	52.6	Chloroplast	TGACv1_486559_6AS: 45,174–47,285
125	TRIAE_CS42_6BL_TGACv1_499376_AA1580390	485	1	5.3	53.3	Chloroplast Cytoplasmic	TGACv1_499376_6BL: 125,626–127,400
126	TRIAE_CS42_6BL_TGACv1_499650_AA1588270	377	1	5.9	40.6	Chloroplast	TGACv1_499650_6BL: 164,606–166,056
127	TRIAE_CS42_6BL_TGACv1_499908_AA1594400	483	1	5.1	53.3	Cytoplasmic	TGACv1_499908_6BL: 62,922–65,065
128	TRIAE_CS42_6BL_TGACv1_500434_AA1604570	464	1	6.2	50.1	Cytoplasmic	TGACv1_500434_6BL:47486–50,072
129	TRIAE_CS42_6BL_TGACv1_500839_AA1610500	490	1	4.9	53.2	Cytoplasmic	TGACv1_500839_6BL: 69,656–71,577
130	TRIAE_CS42_6BL_TGACv1_502282_AA1624090	484	1	5.3	53.2	Cytoplasmic	TGACv1_502282_6BL: 7757–9575
131	TRIAE_CS42_6BS_TGACv1_513285_AA1637340	496	1	5.3	52.3	Chloroplast	TGACv1_513285_6BS: 53,840–59,465
132	TRIAE_CS42_6BS_TGACv1_513359_AA1638830	478	1	5	51.8	Chloroplast Cytoplasmic	TGACv1_513359_6BS: 87,396–89,178
133	TRIAE_CS42_6BS_TGACv1_513952_AA1652850	462	1	6.1	49.1	Mitochondrial Chloroplast	TGACv1_513952_6BS: 89,206–91,232
134	TRIAE_CS42_6BS_TGACv1_514318_AA1658270	493	1	4.8	52.3	Cytoplasmic Chloroplast	TGACv1_514318_6BS: 26,417–28,435
135	TRIAE_CS42_6DL_TGACv1_526838_AA1693090	511	1	10.3	57.2	Nuclear	TGACv1_526838_6DL: 83,730–85,630
136	TRIAE_CS42_6DL_TGACv1_526838_AA1693100	527	1	5.5	57.5	Cytoplasmic	TGACv1_526838_6DL: 91,545–93,815
137	TRIAE_CS42_6DL_TGACv1_527354_AA1702670	479	1	5.8	51.7	Chloroplast PlasmaMembrane Cytoplasmic	TGACv1_527354_6DL: 31,226–34,990
138	TRIAE_CS42_6DL_TGACv1_528544_AA1714910	459	1	5.8	49.6	Cytoplasmic	TGACv1_528544_6DL: 22,658–24,474
139	TRIAE_CS42_6DL_TGACv1_528747_AA1715930	388	1	5.2	43.7	Cytoplasmic	TGACv1_528747_6DL: 19,746–21,729
140	TRIAE_CS42_6DL_TGACv1_529217_AA1717790	537	2	6.7	59	Mitochondrial	TGACv1_529217_6DL: 7806–10,055
141	TRIAE_CS42_6DS_TGACv1_542680_AA1727420	511	1	7.3	55.4	Mitochondrial	TGACv1_542680_6DS: 110,916–113,054
142	TRIAE_CS42_6DS_TGACv1_542696_AA1728130	480	1	5.5	52.3	Chloroplast Cytoplasmic	TGACv1_542696_6DS: 29,236–31,123
143	TRIAE_CS42_6DS_TGACv1_543630_AA1742350	484	2	5.3	53	Chloroplast Cytoplasmic	TGACv1_543630_6DS: 21,813–23,926
144	TRIAE_CS42_6DS_TGACv1_543780_AA1744110	496	1	4.8	52.8	Cytoplasmic Chloroplast	TGACv1_543780_6DS: 23,657–25,745
145	TRIAE_CS42_7AL_TGACv1_556001_AA1752070	731	2	8.8	76.5	PlasmaMembrane Mitochondrial	TGACv1_556001_7AL: 5064–14,615
146	TRIAE_CS42_7AL_TGACv1_556001_AA1752080	460	2	5.5	50.7	Chloroplast	TGACv1_556001_7AL: 63,295–65,765
147	TRIAE_CS42_7AL_TGACv1_556054_AA1753810	488	1	5.7	53.3	Cytoplasmic Chloroplast	IWGSC_CSS_7AL_4383366: 4–1333
148	TRIAE_CS42_7AL_TGACv1_556712_AA1769470	419	1	6	45	Chloroplast PlasmaMembrane Cytoplasmic	TGACv1_556712_7AL: 76,829–78,415
149	TRIAE_CS42_7AL_TGACv1_558513_AA1793890	449	1	5.5	49.3	PlasmaMembrane	TGACv1_558513_7AL: 9716–12,339
150	TRIAE_CS42_7AL_TGACv1_559924_AA1801280	467	2	6.1	49.9	Chloroplast PlasmaMembrane	TGACv1_559924_7AL: 15,613–17,418
151	TRIAE_CS42_7AS_TGACv1_570575_AA1837870	507	1	5.5	54.7	Cytoplasmic	TGACv1_570575_7AS: 49,636–52,320
152	TRIAE_CS42_7AS_TGACv1_571539_AA1848450	469	1	5.5	49.9	Chloroplast	TGACv1_571539_7AS: 11,854–13,474
153	TRIAE_CS42_7AS_TGACv1_573368_AA1852750	442	1	5.6	47.6	Chloroplast Mitochondrial	TGACv1_573368_7AS: 816–2486
154	TRIAE_CS42_7BL_TGACv1_576822_AA1856120	402	1	6.9	44	Chloroplast	TGACv1_576822_7BL: 170,238–172,395
155	TRIAE_CS42_7BL_TGACv1_576994_AA1862270	463	1	5.4	50.9	Cytoplasmic PlasmaMembrane Mitochondrial	TGACv1_576994_7BL: 67,842–69,505
156	TRIAE_CS42_7BL_TGACv1_577254_AA1870230	252	1	4.9	26.9	Cytoplasmic Chloroplast Extracellular	TGACv1_577254_7BL: 76,415–77,415
157	TRIAE_CS42_7BL_TGACv1_577547_AA1878460	420	1	7.2	45.2	PlasmaMembrane Chloroplast	TGACv1_577547_7BL: 29,086–30,601
158	TRIAE_CS42_7BL_TGACv1_579457_AA1907470	484	1	5.2	52.7	Chloroplast	TGACv1_579457_7BL: 41,356–43,187
159	TRIAE_CS42_7BS_TGACv1_591871_AA1924040	397	1	5.1	29.3	Cytoplasmic Chloroplast	TGACv1_591871_7BS: 76,246–80,992
160	TRIAE_CS42_7BS_TGACv1_592186_AA1932820	529	1	5	56.5	Chloroplast Cytoplasmic	TGACv1_592186_7BS: 90,491–92,505
161	TRIAE_CS42_7BS_TGACv1_592546_AA1940110	482	1	6.1	52	PlasmaMembrane Mitochondrial	TGACv1_592546_7BS: 65,246–67,190
162	TRIAE_CS42_7BS_TGACv1_593204_AA1949410	497	1	5.3	53.4	Chloroplast	TGACv1_593204_7BS: 13,157–15,085
163	TRIAE_CS42_7BS_TGACv1_593321_AA1950440	465	1	5.8	50	PlasmaMembrane	TGACv1_593321_7BS: 38,716–40,476
164	TRIAE_CS42_7BS_TGACv1_593432_AA1951550	470	1	5.8	51.4	Chloroplast	TGACv1_593432_7BS: 27,150–29,205
165	TRIAE_CS42_7DL_TGACv1_603213_AA1978480	481	1	5.5	52.2	Chloroplast	TGACv1_603213_7DL: 60,111–61,985
166	TRIAE_CS42_7DL_TGACv1_603403_AA1982990	421	1	5.4	43.7	Chloroplast PlasmaMembrane	TGACv1_603403_7DL: 64,856–66,500
167	TRIAE_CS42_7DL_TGACv1_603951_AA1991550	458	1	5.3	49.8	Chloroplast PlasmaMembrane	TGACv1_603951_7DL: 21,366–23,048
168	TRIAE_CS42_7DL_TGACv1_603951_AA1991560	453	1	5.3	49.1	Chloroplast PlasmaMembrane	TGACv1_603951_7DL: 27,012–28,816
169	TRIAE_CS42_7DL_TGACv1_604766_AA2001560	438	1	5.6	47	Mitochondrial Chloroplast	TGACv1_604766_7DL: 35,976–38,150
170	TRIAE_CS42_7DS_TGACv1_621774_AA2025670	478	1	5.2	50.8	Chloroplast	TGACv1_621774_7DS: 76,896–78,775
171	TRIAE_CS42_7DS_TGACv1_622710_AA2044230	489	2	5.3	54.5	Cytoplasmic	TGACv1_622710_7DS: 15,926–17,908
172	TRIAE_CS42_7DS_TGACv1_623144_AA2050000	447	1	5.8	48.4	Cytoplasmic Chloroplast	TGACv1_623144_7DS: 33,046–34,980
173	TRIAE_CS42_7DS_TGACv1_624130_AA2059090	488	1	5.9	52.5	Mitochondrial PlasmaMembrane	TGACv1_624130_7DS: 15,066–16,815
174	TRIAE_CS42_7DS_TGACv1_626811_AA2066910	480	1	5.7	50.7	PlasmaMembrane Chloroplast	TGACv1_626811_7DS: 3831–5558
175	TRIAE_CS42_U_TGACv1_642463_AA2118110	489	1	6.2	52.4	Chloroplast	TGACv1_642463_U: 55,580–57,595
176	TRIAE_CS42_U_TGACv1_642555_AA2119560	296	1	5.3	31.8	Chloroplast Cytoplasmic	TGACv1_642555_U: 31,767–32,735
177	TRIAE_CS42_U_TGACv1_642847_AA2124040	479	1	5.9	51.5	Chloroplast PlasmaMembrane	TGACv1_642847_U: 26,547–28,358
178	TRIAE_CS42_U_TGACv1_644603_AA2140590	673	1	7.6	72.7	Chloroplast	TGACv1_644603_U: 19,926–22,260
179	TRIAE_CS42_U_TGACv1_658309_AA2151750	368	1	8.3	40.7	Chloroplast	TGACv1_658309_U: 1–1355

### Phylogenetic analysis of UGTs in wheat

**Fig. 1:**
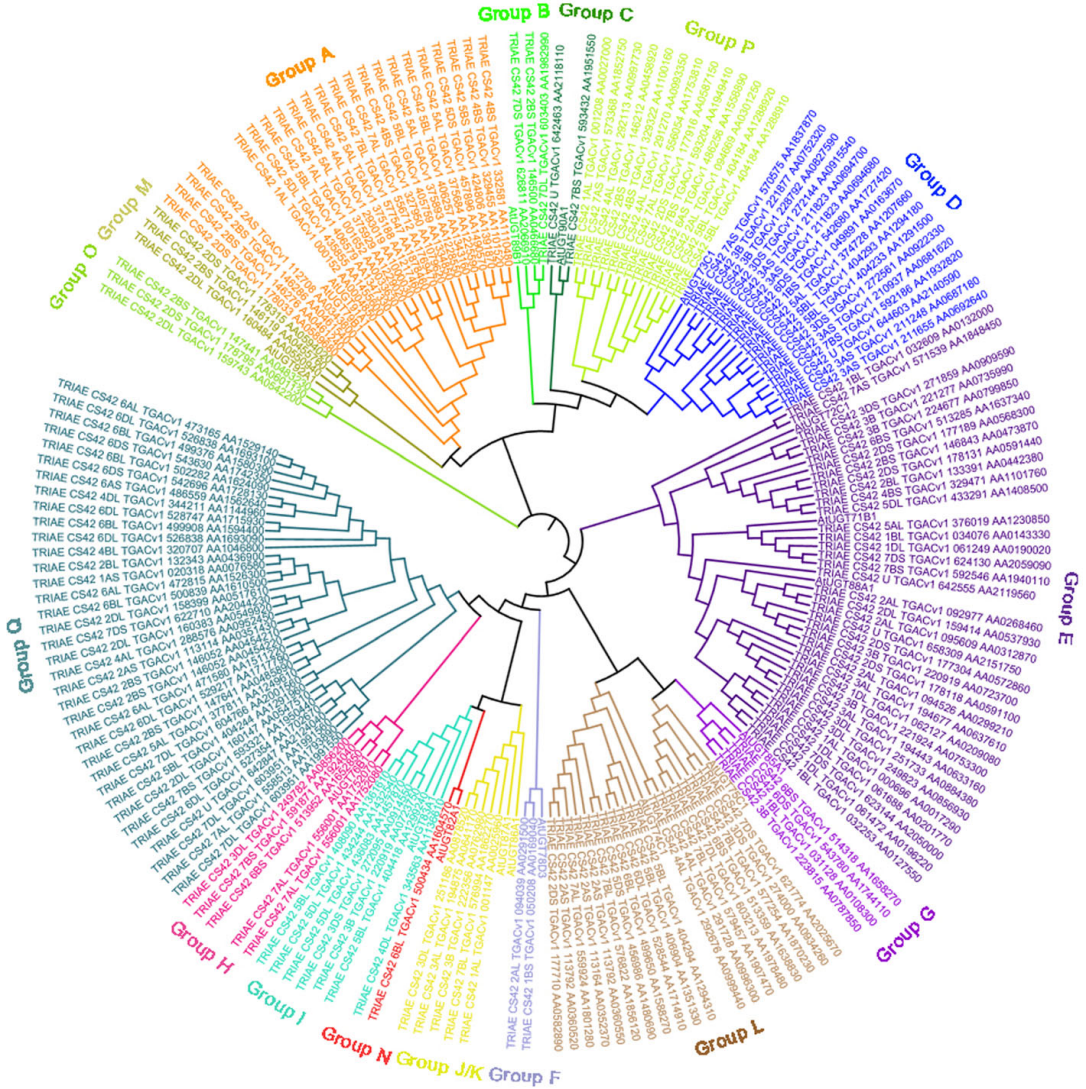
Phylogenetic analysis of bread wheat *UGT* family genes. The MUSCLE and MEGA 7 softwares were used for the sequence alignment and construction of the phylogenetic tree using the full length sequences of 179 wheat UGTs and 18 Arabidopsis UGTs

The identified UGTs were subjected to phylogenetic analysis to see their grouping pattern and genetic relationships based on the 18 Arabidopsis UGTs sequences (Additional file 3: Table S2) [20, 22]. The wheat UGTs were clustered into 16 major phylogenetic groups, with a lack of Arabidopsis conserved phylogenetic group K (Fig. 1). The 14 UGT groups (A-N) described initially in Arabidopsis are considered as conserved groups, and all these groups except group K were found in this study [47]. The number of UGTs in each group varied, as group E, the largest of the groups, contained 37 UGT members, while group N, the smallest of the groups, had only one member. The three new groups identified in our study were O, P and Q containing 3, 13 and 36 UGT members, respectively.

### Genome distribution of wheat *UGT* genes

**Fig. 2:**
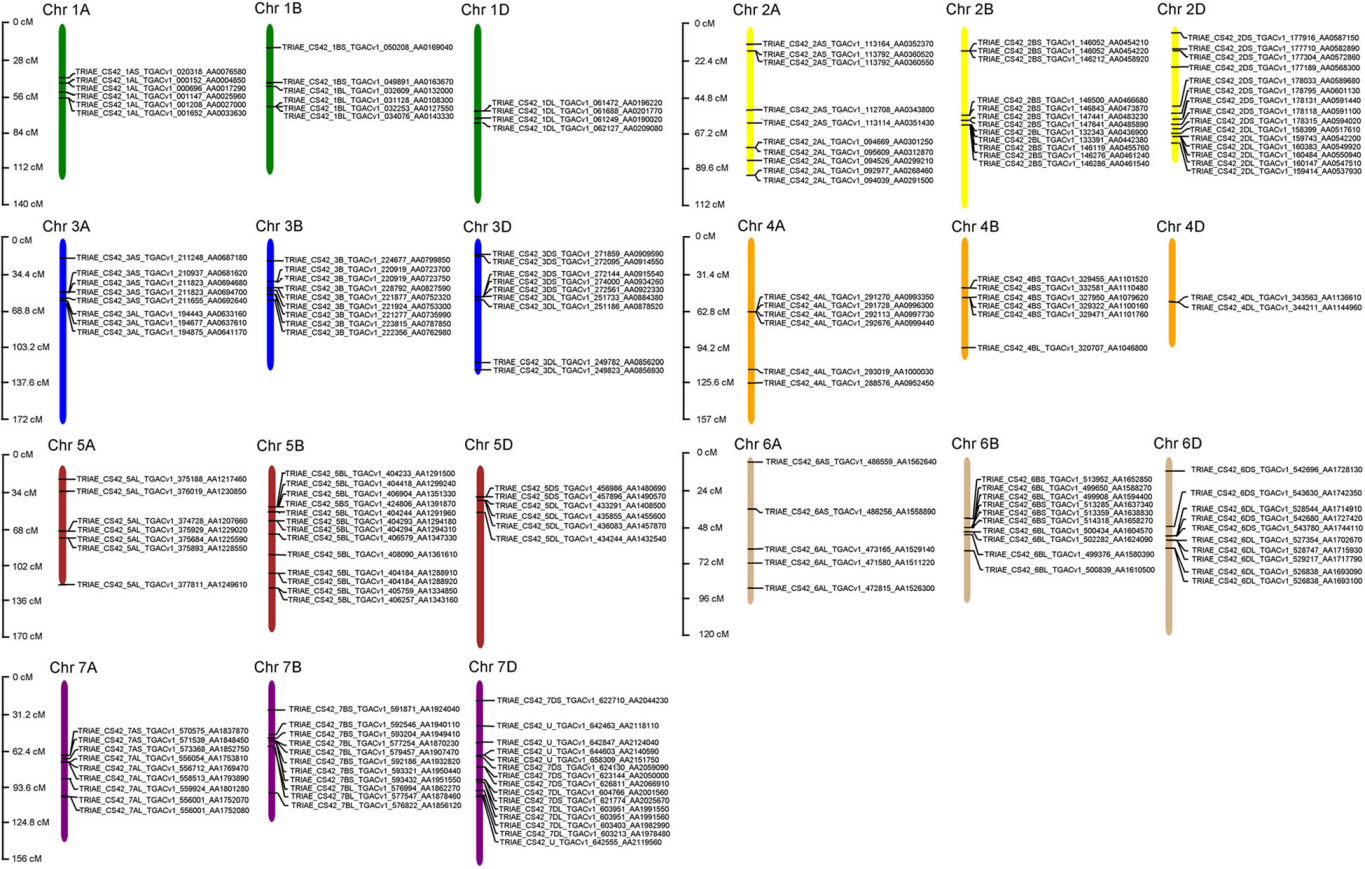
Chromosome distribution of 179 wheat *UGT* genes. The wheat *UGTs* were distributed among seven chromosomes and each of the 3 sub-genomes A, B and D. The different colored bars represent a chromosome and the name of each chromosome along with its sub-genome is mentioned, on top of each bar

Based on the current wheat genome annotation information, the genetic mapping of *UGT* genes on wheat chromosomes was further investigated (Fig. 2). A total of 51, 67, and 61 *UGT* genes were non-randomly distributed in the A, B and D sub-genomes respectively (Table 1; Fig. 2). The number of *UGTs* varied from a minimum of 2 *UGTs* per chromosome to a maximum of 15 *UGTs* per chromosome among all the sub-genomes. Within the sub-genome A, chromosomes 6 and 2 had the minimum (5) and maximum (10) number of *UGTs*, respectively, and within sub-genome B, chromosomes 1 and 4 had the minimum (6) *UGTs* each, and its chromosome 5 had the maximum number (13) of *UGTs*.

### Structure of wheat *UGT* genes

**Fig. 3:**
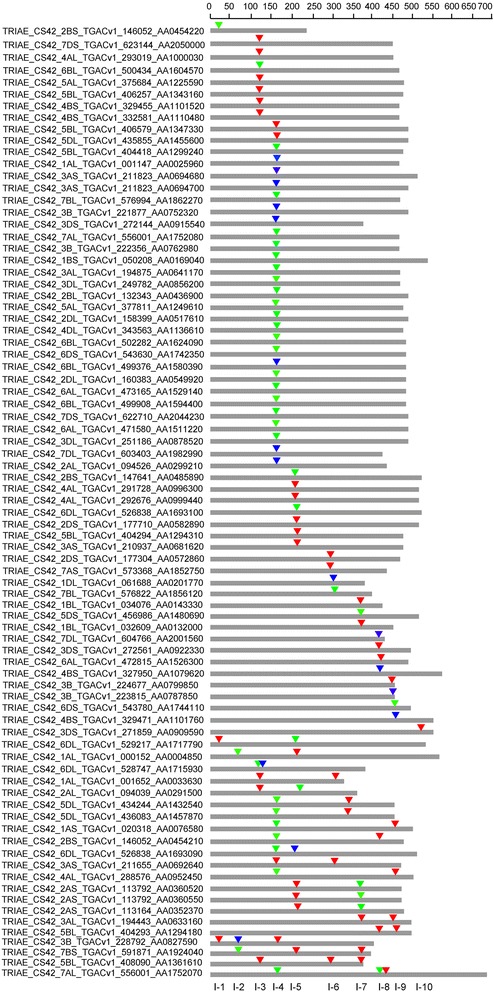
Distribution of introns among 81 wheat *UGT* genes. The map represents the intron positions (inverted triangles) and phases (different colors of the inverted triangles) on the amino acid (thick grey lines) residues encoded by the *UGT* genes. The red, green and blue colors represent the 0, 1 and 2 phases of introns, respectively. The scale on top represents the amino acid count of the *UGT* genes, and the numbers on the bottom represent the identity of each intron

The exon-intron structure is important to know the possibility of existence of alternative isoforms of a gene product that if so, can contribute to the complexity of cellular constitution and compartmentalization. The *UGTs* identified in this study were searched for intron existence, intron size and phases of introns (Additional file 4: Table S3). Among the 179 *UGT* genes identified in this study, 81 *UGTs* (44.5%) contained introns and among the intron containing *UGTs* 60, 17 and 4 had 1, 2 and 3 introns, respectively (Additional file 4: Table S3). After mapping the introns to the amino acid sequence alignment, at least 10 intron insertion events numbered I-1 to I-10, as per their position in the protein sequence, were observed (Fig. 3). The intron (I-4) was the most widespread intron found across 38 sequences of wheat *UGTs* spread across groups A, B, D, E, F, G, H, I, J and Q. The phylogenetic group Q sequences had the highest number (27) of intron insertions, but only 6 different types of introns were found in this group; on the other hand, group A and D shared the highest number (7) of different types of intron insertions (Additional file 4: Table S3). A variable number of intron phases were observed for the UGT protein sequences showing abundance of 0 and 1 phases and scarcity of introns in phase 2. The most abundant phase was 0 (48%), followed by phase 1 (42%) and only 15% were in phase 2 (Additional file 4: Table S3).

### Expression profiles of wheat *UGT* genes in various tissues at different developmental stages

**Fig. 4:**
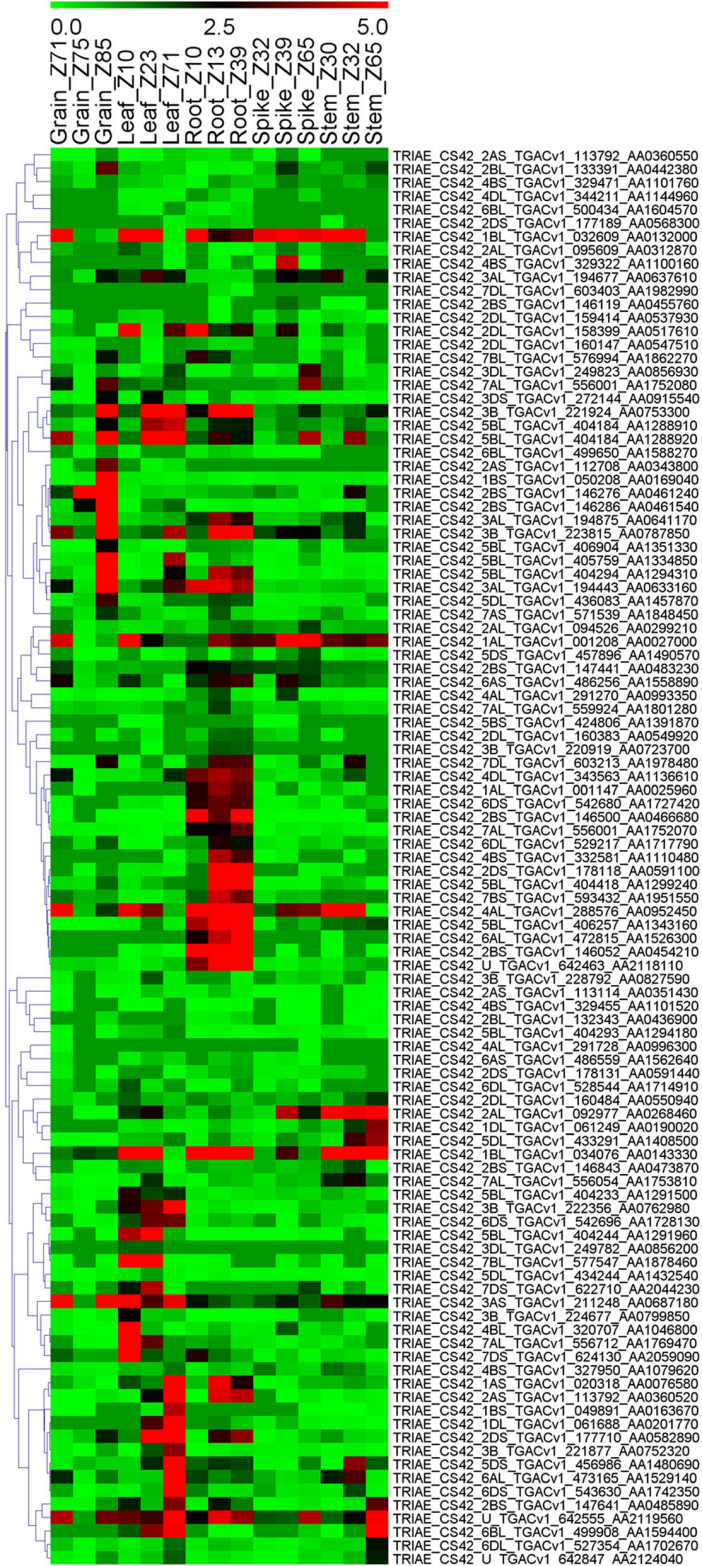
Expression profiles of wheat *UGT* genes in five different tissues at three different developmental stages. The different colors represent the abundance of the transcripts. The developmental stages are denoted using the Zadoks scale

To study the expression profiles of *UGT* genes during the life cycle of the wheat plant, the relative expression of *UGT* genes in the root, shoot, leaf, spike and grains each at three developmental stages were analyzed as per the Zadoks scale [44]. Using the online high-throughput RNA sequences data, the expression profiles of probes representing 109 wheat *UGT* genes were found and were further depicted as a heat map (Fig. 4; Additional file 5: Table S4). The specific expression profile at different developmental stages revealed that most of the genes were expressed at a specific time in each tissue. Among all only nine genes showed extensive expression in almost all of the tissues but not in all the developmental stages and two of these genes *TRIAE_CS42_1BL_TGACv1_032609_AA0132000* and *TRIAE_CS42_4AL_TGACv1_288576_AA0952450* showed the highest expression level in most of the tissues. The expression of most *UGT* genes varied in each organ at different developmental time course, like in grain, leaf, spike and stem the highest expression occurred at the Z85, Z71, Z39 and Z65 stages, respectively (Fig. 4). It was also noted that the highest number of genes were expressed in roots followed by leaves, stem, grains and spikes. None of the genes, except one in spikes, two in stem and three in roots, showed the highest expression in all three developmental stages of these organs. Over all approximately 57% *UGTs* were showing relatively high expression in the life cycle of the wheat plant based on this data (Fig. 4).

### Expression profiles of wheat *UGT* genes under *Fusarium graminearum* treatment

**Fig. 5:**
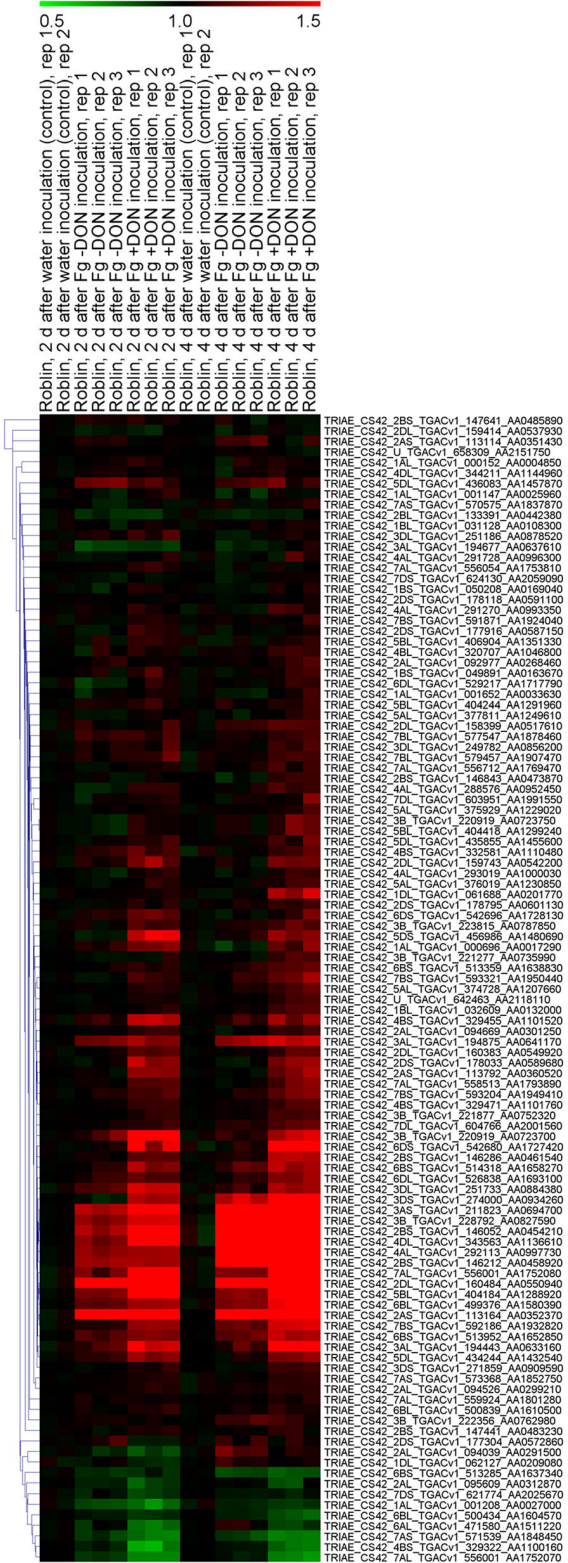
Relative expression profiles of wheat *UGT* genes during Fg-DON and Fg + DON treatment. The wheat spikes were inoculated with the *F. graminearum* strain that did not produce DON (Fg-DON) as well as with the *F. graminearum* strain that produced DON (Fg + DON), with water as the control. Relative expression potential of *UGT* genes is represented by the scale of different colors. The red or green colors represent the higher or lower relative abundance, respectively, of each transcript in each sample

In the present study, expression patterns of the *UGT* genes in response to *F. graminearum* strains producing DON or not after 2 and 4 days after inoculation were investigated using the online Affymetrix wheat array data (GSE54554) to study the roles of *UGT* genes in response to FHB resistance. The expression profile of *UGT* genes at 2 and 4 days after infection (DAI) compared to the control plants showed a differential expression pattern under infected conditions (Fig. 5; Additional file 6: Table S5). One of the most remarkable observations was that the number of genes showing extensive expression during *F. graminearum* stress producing DON was almost double that of the number of genes expressed during *F. graminearum* stress without DON at both 2 DAI and 4 DAI. Among all only 10 genes showed relative higher up-regulation at all circumstances of 2 and 4 DAI except for the control plants, while on the other hand 5 genes were clearly down regulated at all circumstances after *F. graminearum* inoculation (Fig. 5).

### Validation of the expression of *UGT* genes by RT-qPCR

**Fig. 6:**
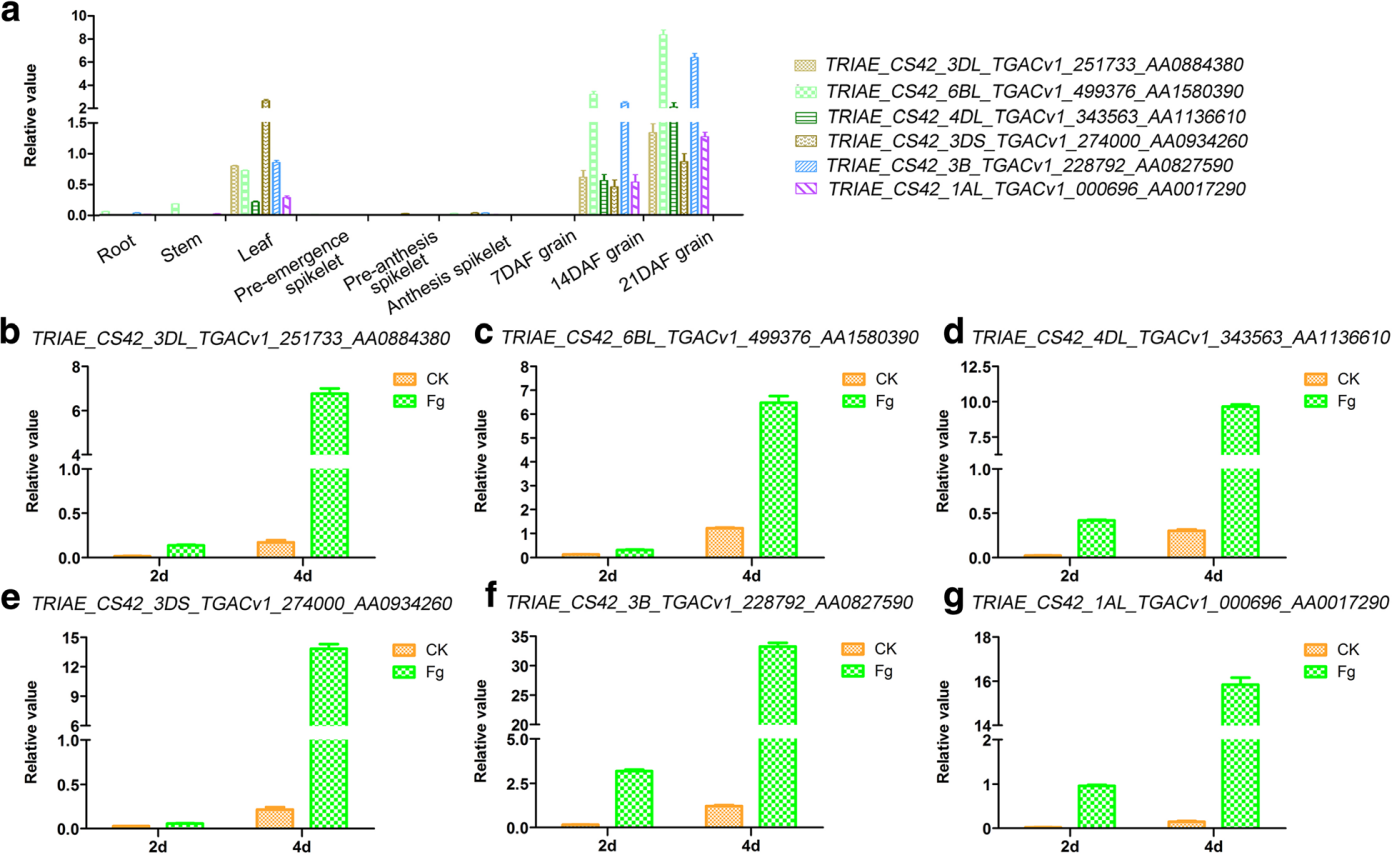
Validation of the expression level of *UGTs* by qRT-PCR analysis. Expression confirmation assay of the six selected *UGT* genes were performed in various wheat tissues at different time intervals (**a**) and under *F. graminearum* treatment (**b**-**g**)

We employed RT-qPCR to validate the expression profile of the *UGT* genes in different tissues as well as during *F. graminearum* inoculation of spikes at different time intervals in wheat. A total of 6 *UGT* transcript sequences were selected to be used for expression profiling in the root, stem, spike and grains and were further employed for expression profiling of the *UGT* genes in *F. graminearum* inoculated spikes. The 6 *UGT* genes were *TRIAE_CS42_4DL_TGACv1_343563_AA1136610*, *TRIAE_CS42_3B_TGACv1_228792_AA0827590*, *TRIAE_CS42_1AL_TGACv1_000696_AA0017290*, *TRIAE_CS42_3DS_TGACv1_274000_AA0934260*, *TRIAE_CS42_3DL_TGACv1_251733_AA0884380* and *TRIAE_CS42_6BL_TGACv1_499376_AA1580390*. The leaf tissues relative to the root and stem showed high expression of the 6 selected genes, in addition to a variable expression of the same genes in the leaf tissues (Fig. 6a). The spikelets at three different developmental stages did not show any noticeable expression of the tested genes (Fig. 6a). The transcript accumulation increased in grains with the development of grain maturity and highest expression was noted at the most mature stage (Fig. 6a). When the spikelets were inoculated with *F. graminearum* at two different time intervals, the results clearly indicated the extensive expression of the selected genes in *F. graminearum* inoculated spikes compared to the control plants (Fig. 6b-g). The gene *TRIAE_CS42_TGACv1_228792_AA0827590* gave the highest relative expression at both time intervals in the *F. graminearum* inoculated spikes compared to the rest of the genes tested (Fig. 6f).

## Discussion

The advent of genome sequencing and the availability of global genomic databases have made it possible to examine complex genomes such as wheat in much greater detail. The identification and validation of FHB resistance genes in wheat is one of the major focuses in the current era of molecular investigations, pertaining to high FHB related economic losses and grain contamination due to DON accumulation. Glycosylation is one of the most important modification and detoxification phenomenon of plant secondary metabolites [15, 48]. Glycosylation, mediated by plants’ indigenous UGTs, in addition to various cellular process and maintenance of cellular homeostasis, also plays a potential role in DON detoxification and FHB resistance. UGTs have been identified and analyzed in a few plant species such as Arabidopsis, flax, maize and cotton; however, they have not been identified on a large scale in wheat except in a few individual reports [14, 15, 20, 22].

In the present study we identified 179 putative family-1 *UGT* genes in wheat based on the Chinese Spring (CS42) reference sequence [40]. The exact number of family-1 *UGT* genes may be alterable in different wheat cultivars as substantial sequence differences such as nucleotides presence/absence are prevalent among cultivars including wheat. The 179 UGT protein sequences were further subjected to multiple sequence alignment and phylogenetic analysis. The multiple sequence alignment of wheat UGTs clearly showed high sequence divergence, especially at the N-terminus, revealing the diverse roles that UGTs play in the life of plants [15]. In this study, the phylogenetic analysis resulted in 16 different (A-Q) phylogenetic groups with one exception. The conserved group K previously described in other studies was absent in our findings, similarly in another study conserved group C was also not found in *Gossypium hirsutum* [15]. The loss of a phylogenetic group during evolution suggests either the loss of function or replacement by other factors [15]. In our study group E contained the highest number (37) of *UGT* genes leading to 21% of the total *UGT* genes identified in wheat. In Arabidopsis, flax and maize group E had 17, 22 and 35 UGT members, respectively, showing the expansion of group E in different plant species [21, 22]. The new groups O, P and Q were found in this investigation. Group Q, first discovered in maize having only 7 members, while in cotton this group was not found; on contrary, our study showed that group Q was not only found but was the 2nd largest group of UGTs in wheat consisting of 36 members [15, 22].

The *UGTs* distribution among the chromosomes showed a dispersion of *UGTs* across all the chromosomes of all the three wheat genomes. A similar pattern of *UGTs* dispersion was also observed in Arabidopsis and among the three species of cotton [15, 47]. Quantitative trait loci (QTLs) for the resistance to *Fusarium* head blight have also been found on all wheat chromosomes, and the most stable QTLs related to FHB resistance are supposed to be located on chromosome 3B, 5A and 6B [49], and here we have shown that these chromosomes have as many as 9, 7 and 10 family-1 *UGTs*, respectively, but the relationship with these QTLs and the exact role of these *UGTs* in resistance to FHB needs to be further studied. Introns, although do not contribute to protein sequences but their position and phases do affect the protein diversity and overall cellular functioning. Introns relative positions can predict certain clues like how genes and their corresponding proteins evolve and further contribute to the diversification of gene families [22, 50]. A total of 10 different intron insertions were identified in this study, while in other crops such as flax and maize 7 and 9 different introns were found, respectively [20, 22]. Among the 179 identified wheat *UGTs*, 55% lacked introns which is in accordance with previous reports on Arabidopsis, flax and maize of which 58%, 55% and 60% lack introns, respectively [20, 22, 47]. In our study, intron 5, found across phylogenetic groups A, B, D, E, F, H, I, J and Q, is considered as the most widespread and oldest intron. Similarly, intron 2 in Arabidopsis found in groups F-K, intron 3 and 4 in flax found in groups F-K, and intron 5 in maize found in groups F-J and N are considered the oldest and most widespread introns [20, 22, 47]. Consistent with other findings, we also observed the abundance of phase 0 and 1 introns compared to phase 2 introns [22]. The existence of different UGTs has been shown in various sub-cellular locations such as cytoplasm, vacuoles, endoplasmic reticulum as well as the membrane [51–54]. DON accumulates in the cytoplasm, plasma membrane and chloroplasts of plant cells [55], and the UGT protein sequences identified in our study also have divergent sub-cellular localization and might lower DON toxicity if confronted in these organelles.

To better understand the roles of the wheat *UGTs* during the life cycle of wheat, we performed an expression analysis of online universal microarray data in certain tissues at different developmental stages. The microarray results have revealed probes that specifically match 61% of the identified wheat *UGTs*, and most of these genes have been expressed at least in a certain tissue during the life cycle of the wheat plant. Similarly, in other crops such as maize and flax it has been shown that 82% and 73% of the corresponding genes showed expression [20, 22]. The genes analyzed in different tissues, as per the microarray results, showed that 13%, 29%, 35%, 9% and 15% of the *UGT* genes displayed extensive overexpression in grains, leaf, root, spikes and stem, respectively, during all the various stages studied. Using selected *UGT* gene sequences, RT-qPCR also revealed a differential expression profile in most of the growth stages in certain wheat tissues, suggesting that the *UGTs* are opting for preferential expression in particular organs during the life cycle of the wheat plant.

FHB is a menace for agriculture crops, especially for wheat growing in the humid regions of the world, and current focus has been placed on understanding the molecular mechanisms behind FHB resistance and the development of germplasms resistant to FHB. It is important to outline the role of the UGTs identified in this study, if any, during the *F. graminearum* incidence that could further be utilized for the development of resistance against *F. graminearum* stress. As many other investigators have previously shown the involvement UGTs in host resistance against FHB both in wheat as well as in barley [33, 37]. In our study, the *F. graminearum* stress responsive genes analyzed using online microarray data revealed some interesting results that were further validated by expression analysis of selected *UGT* genes using RT-qPCR. The wheat spikes were inoculated with a mutated *F. graminearum* strain that does not produce DON (Fg-DON) as well as with an *F. graminearum* strain that produces DON (Fg + DON), with water as the control. An average of 27 and 59% of the *UGT* genes were up regulated after Fg-DON and Fg + DON inoculation, respectively, compared with the control at 2 DAI. On the other hand, an average of 32 and 69% of the *UGT* genes displayed up-regulation in the Fg-DON and Fg + DON inoculated plants, respectively, at 4 DAI compared to the control plants. The up-regulation of a high number of *UGTs* during Fg + DON inoculation is an indication of the wheat indigenous UGTs based DON responsive defense mechanism against FHB. The data also clearly show an extensive up-regulation of a high number of genes on the 4th day after Fg + DON inoculation, showing an increase in response as DON accumulation increases. These results were further confirmed through RT-qPCR amplification of 6 selected *UGT* genes, where the highest expression was evident at 4 DAI. These genes and validation of the microarray data using a resistant genotype such as Sumai 3 will be the subject of our ongoing research to further dissect the wheat indigenous defense mechanisms and to identify the resistance source underlying *F. graminearum* infection and DON detoxification.

## Conclusions

This study gave a useful insight into the phylogenetic structure, distribution, and expression patterns of family-1 UDP glycosyltransferases of wheat. The results also offer a foundation for future work aimed at elucidating the molecular mechanisms underlying *F. graminearum* resistance and DON detoxification in one of the world’s most important cereal crops.

## Additional files

Additional file 1: **Table S1.** List of the selected *UGT* genes and their primers sequences used for the RT-qPCR expression assay. (XLSX 9 kb)

Additional file 2: **Figure S1.** The abundance of wheat *UGT* genes as per their amino acid sequence sizes. (JPG 38 kb)

Additional file 3: **Table S2.** List of the Arabidopsis *UGT* genes used in this study for the identification of the phylogenetic groups. (XLSX 9 kb)

Additional file 4: **Table S3.** The intron information of the wheat *UGT* genes. (XLSX 35 kb)

Additional file 5: **Table S4.** Expression data of *UGT* genes in five different tissues at different developmental stages. (XLSX 20 kb)

Additional file 6: **Table S5.** Expression data of *UGT* genes after *F. graminearum* inoculation. (XLSX 35 kb)

## Abbreviations

DAI: Days after infection
DON: Deoxynivalenol
Fg: *Fusarium graminearum*
FHB: *Fusarium* head blight
GT: Glycosyltransferase enzymes
PSPG: Plant secondary product glycosyltransferase
QTL: Quantitative trait loci
UGT: UDP glycosyltransferases
