# Supplementary figures and images for: Genome-wide analysis of family-1 UDP glycosyltransferases (UGT) and identification of UGT genes for FHB resistance in wheat (Triticum aestivum L.)
Source: BMC Plant Biol. 2018 Apr 19;18:67. doi: 10.1186/s12870-018-1286-5 (PMC5909277; doi:10.1186/s12870-018-1286-5)

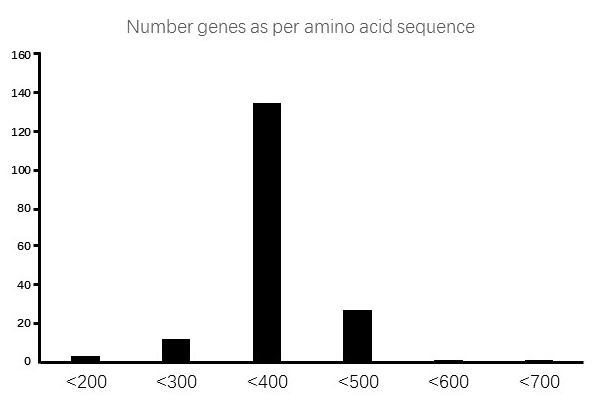

Supplement: Supplementary file 2 — Figure S1. The abundance of wheat UGT genes as per their amino acid sequence sizes. (JPG 38 kb) [file 12870_2018_1286_MOESM2_ESM.jpg]
